# Reactive oxygen species induced by therapeutic CD20 antibodies inhibit natural killer cell-mediated antibody-dependent cellular cytotoxicity against primary CLL cells

**DOI:** 10.18632/oncotarget.8769

**Published:** 2016-04-16

**Authors:** Olle Werlenius, Johan Aurelius, Alexander Hallner, Ali A. Akhiani, Maria Simpanen, Anna Martner, Per-Ola Andersson, Kristoffer Hellstrand, Fredrik B. Thorén

**Affiliations:** ^1^ Department of Hematology, Sahlgrenska University Hospital, Gothenburg, Sweden; ^2^ TIMM Laboratory, Sahlgrenska Cancer Center, University of Gothenburg, Gothenburg, Sweden; ^3^ Department of Medicine, Södra Älvsborgs Hospital, Borås, Sweden

**Keywords:** monoclonal antibodies, reactive oxygen species, immunotherapy, NK cells, NOX2

## Abstract

The antibody-dependent cellular cytotoxicity (ADCC) of natural killer (NK) cells is assumed to contribute to the clinical efficacy of monoclonal antibodies (mAbs) in chronic lymphocytic leukemia (CLL) and other hematopoietic malignancies of B cell origin. We sought to determine whether reactive oxygen species (ROS)-producing monocytes regulate the ADCC of NK cells against primary CLL cells using anti-CD20 as the linking antibody. The monoclonal CD20 antibodies rituximab and ofatumumab were found to trigger substantial release of ROS from monocytes. Antibody-exposed monocytes induced NK cell apoptosis and restricted NK cell-mediated ADCC against autologous CLL cells. The presence of inhibitors of ROS formation and scavengers of ROS preserved NK cell viability and restored NK cell-mediated ADCC against primary CLL cells. We propose that limiting the antibody-induced induction of immunosuppressive ROS may improve the anti-leukemic efficacy of anti-CD20 therapy in CLL.

## INTRODUCTION

Subsets of cytotoxic leukocytes, including myeloid cells and NK cells, carry different Fcγ-receptors (FcγR) and may thus attach the Fc portion of IgG antibodies to exert cytotoxicity against cells expressing a specific antigen [1]. ADCC is assumed to contribute to the clinical benefit of antibodies against CD20, such as rituximab (RTX) and ofatumumab (OFA), in B cell malignancies, and recent studies support that FcγR^+^ NK cells are pivotal for the anti-leukemic efficacy [2–4]. Several authors report, however, that the NK cell population is functionally deficient in CLL [5, 6], and defining mechanisms of immunosuppression in this disease may be helpful in understanding the immunobiology of CLL and may have therapeutic implications.

Myeloid cells, such as monocytes and neutrophils, harbor the NOX2-containing NADPH oxidase, which is a multi-component enzyme complex that transfers electrons to molecular oxygen to generate superoxide anion and other ROS. These highly reactive compounds are pivotal in elimination of ingested microbes but are also potent immunoregulatory compounds that suppress lymphocyte-mediated immunity [7], and NOX2-derived ROS are assumed to contribute in preventing autoimmune manifestations [8, 9]. In addition, extracellular ROS, released from normal myeloid cells [10] as well as from myeloid-derived suppressor cells [11], tumor-associated macrophages [12] and malignant myeloid cells [13–16] have been proposed to contribute to cancer-related immunosuppression in several malignancies, including leukemia.

In B cell malignancies, myeloid cells have been proposed to reduce NK cell-mediated cytotoxicity against CD20-opsonized targets by metalloproteinase-mediated shaving [17] or by extraction of the antibody-bound CD20 antigen by trogocytosis [18]. In addition, it was recently reported that CD20 antibodies triggered neutrophils to release extracellular ROS [19], but the impact of antibody-triggered ROS on NK cell ADCC is not known. For this study, we hypothesized that reducing myeloid cell-derived ROS may improve the anti-leukemic efficacy of CD20 antibodies and thus investigated the impact of human NOX2^+^ monocytes on ADCC against human primary CLL cells in the presence or absence of anti-CD20 and anti-oxidative compounds. Our results suggest that monocytes produce and release significant amounts of ROS in response to both RTX and OFA. The ROS production limited the NK cell-mediated ADCC against primary leukemic cells, as NK cell ADCC could be partially restored by anti-oxidative agents. We propose that monocyte-mediated suppressive mechanisms, including trogocytosis and release of immunosuppressive ROS, may limit the benefit of anti-CD20 mAbs, and that anti-oxidative strategies may be of value to enhance the clinical efficacy of anti-CD20 therapy.

## RESULTS AND DISCUSSION

**Figure 1:**
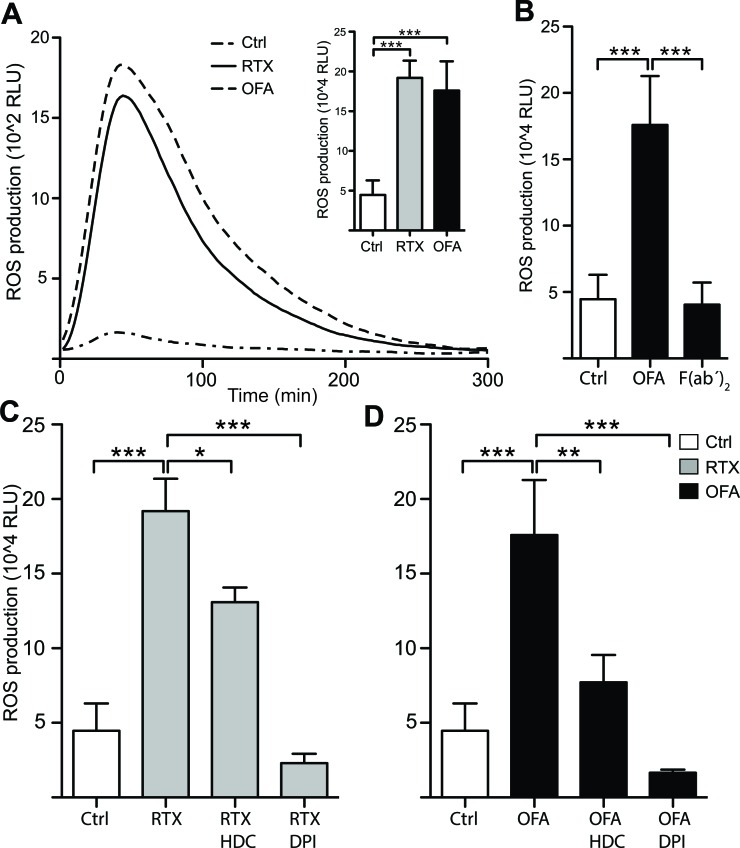
Rituximab and Ofatumumab triggered ROS production by monocytes Monocytes derived from healthy blood donors were continuously assessed for extracellular ROS production by chemiluminescense in the presence of CLL cells (Mo:CLL ratio 2:1) and the presence or absence of CD20 mAbs (10μg/ml). **A.** shows a representative kinetic graph, while the inset column diagram shows total ROS production (Area Under Curve; AUC; *n* = 4). **B.** shows total ROS production in presence and absence of OFA (10μg/ml) and OFA-derived F(ab')_2_ fragments (10μg/ml). **C.**, **D.** Total ROS production in presence and absence of the ROS formation inhibitors histamine dihydrochloride (HDC;100μM) and diphenylene iodonium chloride (DPI; 3μM) (*n* = 4). Statistical significance for all figures was determined by one-way ANOVAs and the Bonferroni post test. (RLU;relative light units) *p < 0.05, **p < 0.01, ***p < 0.001.

In a first series of experiments we investigated whether CD20 mAbs mount an oxidative response from monocytes. As shown in Figure 1, RTX and OFA triggered a pronounced release of extracellular ROS from purified monocytes. The ROS production was mediated by NOX2, as inhibitors of this enzyme, HDC and DPI [20, 21], prevented the antibody-induced ROS formation. To clarify the role of Fc receptors and for the antibody-induced formation of ROS, we generated F(ab’)_2_ fragments of ofatumumab and found that F(ab’)_2_ fragments did not induce ROS, thus implying that monocytes produce ROS upon attaching to the FC-part of anti-CD20 Ab (Figure 1A).

**Figure 2:**
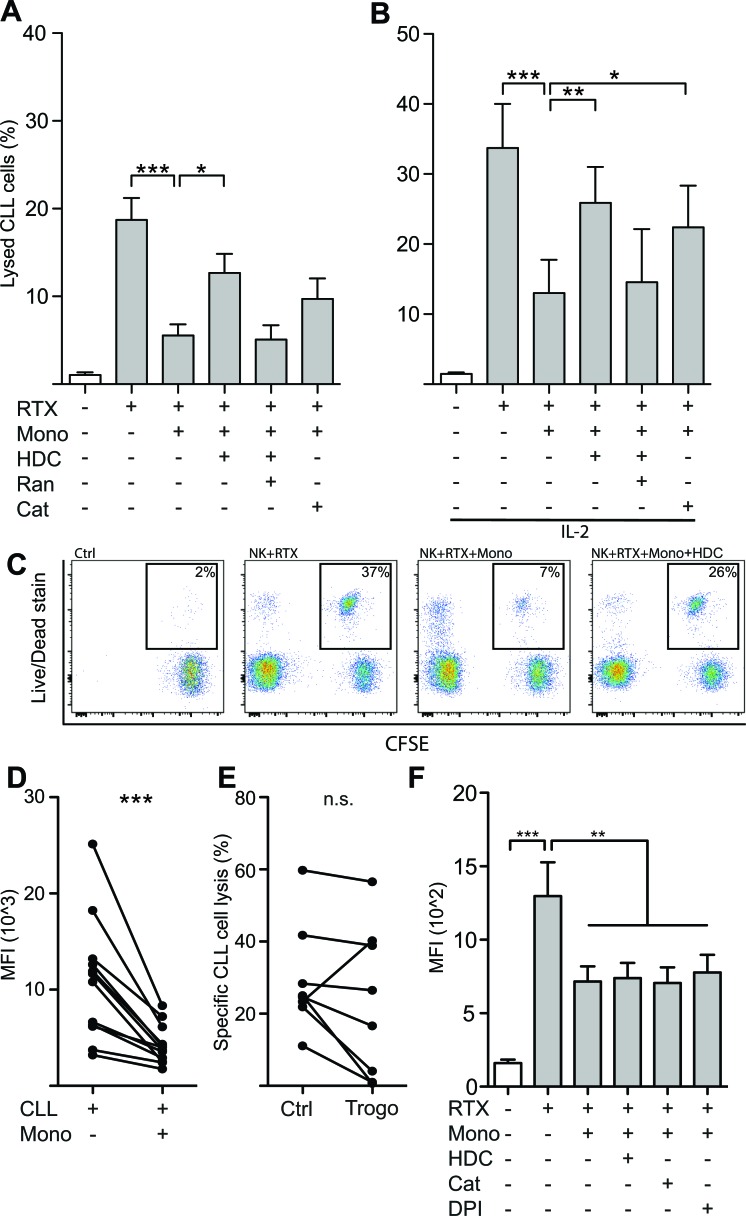
Monocytes restricted NK cell ADCC against autologous leukemic cells by production of ROS **A.**, **B.** NK cells and CFSE-labeled CLL cells were co-cultured for four hours in the presence or absence of autologous monocytes at an NK:Mo:CLL-ratio of 2:2:1 and IL-2 (500IU/ml), rituximab (10μg/ml), HDC (100μM), ranitidine (Ran; 100μM) or catalase (Cat; 200IU/ml). ADCC was inhibited by the presence of monocytes, but largely restored by anti-oxidative agents HDC or catalase. (*n* = 5-7). **C.** Representative dot-plot depicting the read-out for lysed leukemic cells of panels A and B. Percentages denote the proportion of lysed leukemic cells, thus staining positive for the Live/Dead stain. **D.** Monocytes were found to decrease the density of surface-bound rituximab on CLL cells, a mechanism referred to as trogocytosis. **E.** NK cell-mediated ADCC of CLL cells previously exposed to monocytes, and thus allowing for antigen removal by trogocytosis, was lowered in 7 out of 8 performed experiments. **F.** Monocyte-mediated trogocytosis was unaffected by addition of anti-oxidative substances (*n* = 4). *p < 0.05, **p < 0.01, ***p < 0.001.

We next determined whether the presence of monocytes interfered with NK cell-mediated killing of autologous CLL cells. In accordance with earlier reports [22, 23], NK cells isolated from CLL patients induced significant CLL cell death in the presence of RTX with only minor cytotoxicity in the absence of a linking antibody. Monocytes failed to exert substantial RTX-dependent cytotoxicity against CLL cells. Instead, NK cell ADCC was strongly reduced in the presence of monocytes (Figure 2A-2B). HDC and the ROS-degrading enzyme catalase both partially restored the diminished ADCC of NK cells. Neither HDC nor catalase affected CLL cell viability or ADCC by NK cells in the absence of monocytes (data not shown). The NK cell-activating cytokine IL-2 augmented RTX-mediated ADCC by NK cells but did not rescue NK cells from ROS-induced inhibition (Figure 2B). Similar results were obtained using OFA in ADCC assays (data not shown).

The incomplete restoration of cytotoxicity by anti-oxidative compounds suggested that additional mechanisms might have contributed to the observed inhibition of ADCC by monocytes. Previous studies have show that monocytes upon interaction with CD20 mAb-opsonized CLL cells may shave off or extract the antibody-antigen complex from the CLL cells, a mechanism known as trogocytosis, thus reducing the amount of antibody bound to the CLL cells and limiting NK cell-mediated ADCC [17, 18]. To address the impact of this inhibitory mechanism, we exposed CD20 mAb-opsonized CLL cells to monocytes and determined the level of bound antibody on CLL cells after 45 minutes of incubation. As shown in Figure 2D, monocytes reduced the amount of RTX bound to CLL cells. To investigate whether this reduction of bound antibody could explain the incomplete restoration of ADCC by antioxidative agents, we removed monocytes from the CLL cells using anti-CD14 beads, re-introduced RTX (10μg/ml) and determined the CLL susceptibility to ADCC. As shown in Figure 2E, monocyte-induced trogocytosis of bound mAbs and antigens caused a slight, reduction of ADCC in 7 out of 8 experiments, though the observed reduction was not statistically significant. The addition of HDC, catalase or DPI did not affect the ability of monocytes to reduce RTX/OFA binding to CLL cells (Figure 2F). The restoration of ADCC observed in the presence of anti-oxidative substances was not due to inhibition of trogocytosis, but it is possible that trogocytosis, at least in part, could explain the incomplete restoration of ADCC by anti-oxidative reagents.

**Figure 3:**
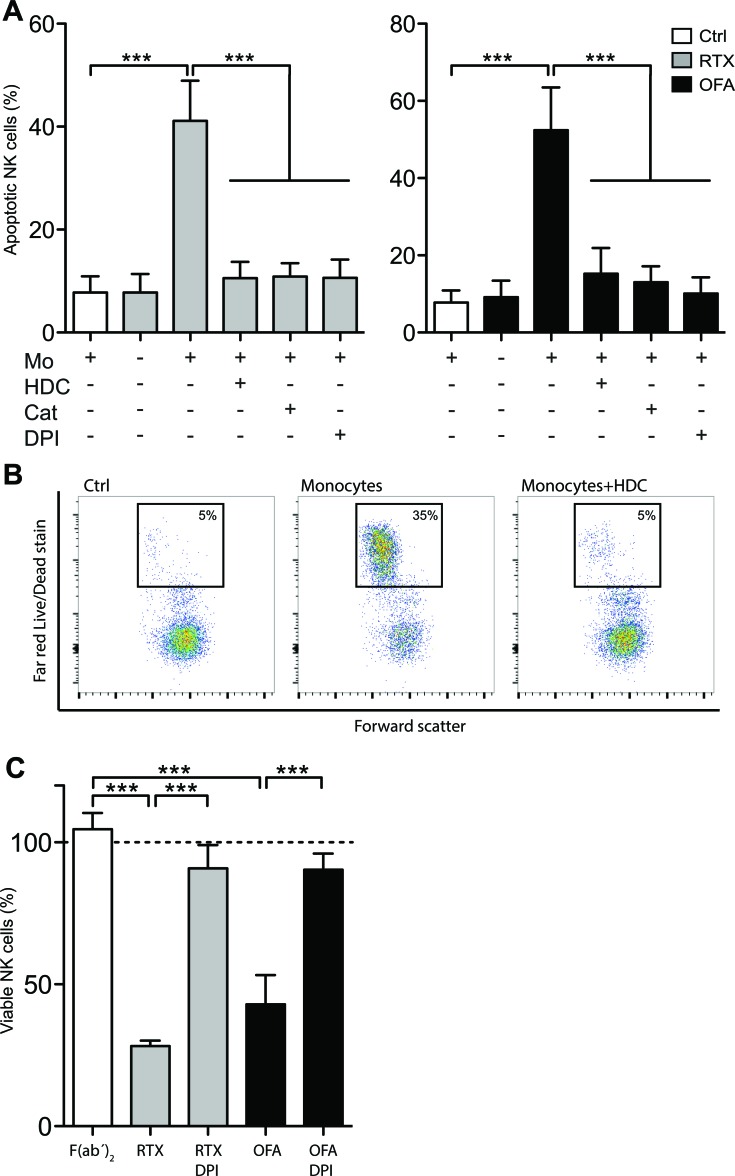
CD20 antibodies induced ROS-dependent NK cell apoptosis **A.** NK cells and monocytes were co-cultured for 18 hours at a Mo:NK-ratio of 1:2 in presence or absence of immobilized, plate-bound RTX (5μg/ml; left) or OFA (5μg/ml; right) and HDC (100μM), catalase (200IU/ml) or DPI (3μM) (*n* = 4). **B.** Representative dot-plot of the read-out for dead NK cells shown in panel A. **C.** PBMCs and PMNs at equal ratios were cultured for 18 hours in the presence or absence of plate-bound RTX, OFA or OFA- derived F(ab’)_2_ (10 μg/ml) and DPI (3μM). Bars show percentage of remaining, viable CD 56^+^CD3^−^ NK cells compared to cultures with no antibody added (dotted line; *n* = 5). ***p < 0.001.

It was previously demonstrated that ROS produced from normal and leukemic myeloid cells trigger programmed cell death in NK cells [7, 14, 24, 25]. We thus investigated whether CD20 antibodies affected NK cell viability in the presence of ROS-producing monocytes. Using immobilized mAbs, which enable efficient cross-linking of Fc receptors and thus enhance the amplitude of transmitted signals, we observed that RTX and OFA induced extensive NK cell death, which was entirely prevented by HDC, DPI or catalase (Figure 3A). Similar results were obtained in experiments using leukocyte proportions aiming to mimic the situation in blood or bone marrow (Figure 3C). These findings support that ROS production by adjacent myeloid cells constitutes a negative regulator of NK cell function and that therapeutic CD20 antibodies reinforce myeloid cell-induced, ROS-mediated immunosuppression, thus reducing the efficiency of the ADCC reaction.

Our results add to a growing body of evidence that proposes a role for myeloid cells in disease progression and maintenance of the leukemic clone in CLL. Burger and co-workers first described nurse-like cells, a population of monocyte-derived CD14^+^ cells that supported the survival of CLL cells *in vitro* [26]. Others reported that patients with CLL frequently harbor myeloid-derived suppressor cells (MDSCs) and that presence of these immunosuppressive, CD14^+^HLA-DR^lo^ monocytes in the peripheral blood is associated with advanced disease and poor prognosis [27, 28]. In addition, Hanna *et al*. recently reported that depletion of monocytes in a mouse model of CLL resulted in significantly lower tumor burden [29]. Taken together, these reports highlight the myeloid compartment as a potential therapeutic target in CLL.

The NOX2 inhibitor HDC is used in conjunction with low-dose IL-2 as relapse-preventive immunotherapy in acute myeloid leukemia (AML), and the clinical efficacy of HDC/IL-2 is pronounced in AML with monocytic differentiation [13]. *In vitro* studies imply that patients with this subset of AML harbor malignant cells with capacity to produce extracellular ROS that inhibit adjacent NK cells [14]. Although CLL cells do not produce ROS, our finding that monocytes derived from patients with CLL are triggered to produce ROS by therapeutic antibodies suggests that a similar immunosuppressive environment may be operative in CLL during treatment with monoclonal antibodies. The anti-CD20-induced ROS-dependent suppression and apoptosis of NK cells induced by monocytes was inhibited by anti-oxidative agents, which translated into improved anti-leukemic efficacy. We propose further studies to clarify whether compounds that rescue NK cells from ROS-induced inactivation may improve the clinical efficacy of mAb-based therapy in CLL.

## MATERIALS AND METHODS

### Isolation of cells

Blood samples from patients with CLL were collected after informed consent. Leukopacks from healthy blood donors were obtained from the Blood Center at Sahlgrenska University Hospital. Peripheral blood mononuclear cells (PBMC) and polymorphonuclear cells (PMN) were isolated using dextran sedimentation followed by density gradient centrifugation and lysis of remaining erythrocytes by deionized water. PBMCs obtained from CLL patients were stained with antibodies, and CD3^−^/CD56^+^ NK cells and CD14^+^ monocytes were FACS-sorted using a BD FACSAria III. For isolation of malignant CLL cells a Bcell (B-CLL) isolation kit was used according to the manufacturer's instructions (Purity > 90%; Miltenyi Biotec). NK cells and monocytes were isolated from blood donor PBMCs by use of the corresponding MACS isolation kits (purity > 95% and 92%, respectively; Miltenyi Biotec).

### Generation of F(ab′)_2_ fragments

F(ab′)_2_ fragments of ofatumumab were prepared by pepsin digestion using Pierce F(ab′)_2_ preparation kit #44988 (Life Technologies) according to instructions provided by the manufacturer. Digestion and purity were confirmed by SDS-PAGE (Life Technologies).

### ROS production

Extracellular ROS production by monocytes was assessed by chemiluminescence as described in detail elsewhere [14]. In brief, 2*10^5^ monocytes were suspended in Krebs-Ringer Glucose buffer supplemented with isoluminol (10 μg/ml) and horseradish peroxidase (4 U/ml). Monocytes were incubated at 37°C in the presence or absence of mAbs (10 μg/ml) and primary CLL cells, and the release of ROS (light emission) was continuously monitored using a BMG FLUOStar Microplate Reader. In some experiments, ofatumumab F(ab′)_2_ fragments were used to define the role of the Fc portion.

### NK cell death

NK cells and monocytes were co-cultured overnight at various ratios (NK cell absolute count 2*10^5^) at 37°C and 5% CO_2_ in the presence or absence of immobilized mAbs (5μg/ml) and anti-oxidative compounds. NK cell death was determined by flow cytometry after staining with a LIVE/DEAD^®^ cell stain kit (Life Technologies). In some experiments leukocyte suspensions with physiologic cell proportions (adding an equal amount of PMNs to isolated PBMCs) were used (RTX and OFA 10 μg/ml). In these experiments, NK cells were identified as CD3^−^CD56^+^ lymphocytes, and fluorescent counting beads were used to determine the number of surviving NK cells.

### ADCC assays

Isolated primary CLL cells were labeled with CFSE (Life Technologies) for traceability. Autologous NK cells, monocytes and CLL cells were co-cultured at a 2:2:1 ratio (absolute counts of 5*10^4^ for NK cells and monocytes and 2.5*10^4^ for CLL cells) in presence or absence of rituximab (10μg/ml) and anti-oxidative compounds and incubated at 37°C and 5% CO_2_. After four hours the cells were stained with a LIVE/DEAD^®^ cell stain and assessed for target cell death by flow cytometry. In experiments with ofatumumab (10μg/ml) NK cells and monocytes derived from healthy donor samples were used.

### Trogocytosis assay

Isolated primary CLL cells were labeled with CellTrace Violet (Life Technologies) and incubated at 37°C and 5 % CO_2_ with rituximab (10 μg/ml) for 30 min. Freshly isolated monocytes and opsonized CLL cells were co-cultured after a 15 s centrifugation (300 g) at a 2:1 ratio in a flat-bottomed 96-well plate for 45 min. In some experiments, monocytes were subsequently removed using IMag CD14 magnetic particles (BD Biosciences). CLL cells were stained with an anti-IgG FITC antibody (Jackson ImmunoResearch) and assessed for RTX-bound CD20 expression by flow cytometry. In some experiments, CLL cells previously exposed to monocytes were subjected to an ADCC assay using freshly isolated NK cells and rituximab (10 μg/ml) as described above.

### Compounds

The following compounds were used: rituximab (RTX; Roche), ofatumumab (OFA; GSK), histamine dihydrochloride (HDC; 100μM), ranitidine (100μM), catalase (200U/ml), interleukin-2 (IL-2; 500U/ml; Chiron) and diphenylene iodonium chloride (DPI; 3μM; Sigma).

### Ethics

This study was approved by the ethical review board of Gothenburg. All experiments were performed in accordance with the Declaration of Helsinki.
